# Influence of Nitrogen Availability on Growth of Two Transgenic Birch Species Carrying the Pine GS1a Gene

**DOI:** 10.3390/plants6010004

**Published:** 2017-01-06

**Authors:** Vadim G. Lebedev, Nina P. Kovalenko, Konstantin A. Shestibratov

**Affiliations:** Branch of Shemyakin and Ovchinnikov Institute of Bioorganic Chemistry of the Russian Academy of Sciences, Science avenue 6, Pushchino, Moscow Region 142290, Russia; nina-kovalenko4@rambler.ru (N.P.K.); schestibratov.k@yandex.ru (K.A.S.)

**Keywords:** *Betula*, chlorophyll, glutamine synthetase, nitrogen fertilization, transgenic birch

## Abstract

An alternative way to increase plant productivity through the use of nitrogen fertilizers is to improve the efficiency of nitrogen utilization via genetic engineering. The effects of overexpression of pine glutamine synthetase (GS) gene and nitrogen availability on growth and leaf pigment levels of two *Betula* species were studied. Untransformed and transgenic plants of downy birch (*B. pubescens*) and silver birch (*B. pendula*) were grown under open-air conditions at three nitrogen regimes (0, 1, or 10 mM) for one growing season. The transfer of the GS1a gene led to a significant increase in the height of only two transgenic lines of nine *B. pubescens*, but three of five *B. pendula* transgenic lines were higher than the controls. In general, nitrogen supply reduced the positive effect of the GS gene on the growth of transgenic birch plants. No differences in leaf pigment levels between control and transgenic plants were found. Nitrogen fertilization increased leaf chlorophyll content in untransformed plants but its effect on most of the transgenic lines was insignificant. The results suggest that birch plants carrying the GS gene use nitrogen more efficiently, especially when growing in nitrogen deficient soil. Transgenic lines were less responsive to nitrogen supply in comparison to wild-type plants.

## 1. Introduction

Nitrogen plays a key role in the growth and development of plants as it is a component of amino acids, chlorophyll, nucleic acids, and growth regulators. This element is the main limiting factor for plant productivity in temperate and boreal forest ecosystems [1]. Forest plantations are also mainly established on poor soils, where nitrogen availability is limited. For this reason, nitrogen fertilizers are used intensively for increasing yield and reducing rotation age in forest plantations [2]. However, synthetic nitrogen fertilizers are expensive. At the same time, 50%–75% of the nitrogen applied to fields are not assimilated by plants and are lost [3], thus contaminating soil, water, and air. An alternative way to increase plant productivity is to improve the efficiency of nitrogen utilization, in particular, by means of genetic engineering.

For this purpose, the incorporation of additional glutamine synthetase (GS) genes is used most frequently. This is due to the fact that GS is a main enzyme for nitrogen assimilation in plants [4], as products of GS/GOGAT cycle (glutamate and glutamine) are precursors for organic compounds in plants [5]. GS genes were transferred into numerous herbaceous plants. However, up to now their effect has been only studied for a single woody plant. A hybrid *Populus tremula* × *P. alba* with a gene for thecytosolic form of pine GS has demonstrated an increased productivity under both greenhouse [6] and field conditions [7]. This gene was used because, the cytosolic form of the enzyme from gymnosperms is active in photosynthetic tissues, in contrast to angiosperms [8]. Results of studies [6,7] suggest that accelerated growth of transgenic trees of the *Populus* hybrid is not only explained by the primary nitrogen assimilation, but also by the reassimilation of ammonium, which is produced during various metabolic processes [7].

Most of the studies on both genetic transformation and influence of nitrogen availability are carried out in species and hybrids of *Populus* [9,10], which are used worldwide on short-rotation plantations. Birch is the most important broad-leaved tree species in the Northern and Eastern Europe forestry [11]. In order to increase productivity of forest plantations the gene of cytosolic GS from *Pinus sylvestris* was transferred into the genome of *Betula* [12]. Transgenic plants were tested for growth rate enhancement in the greenhouse [13] and resistance to phosphinothricin treatment [14]. As an extension of the evaluation of these plants, in the present work we estimated the effect of the pine GS1a gene expression on the growth and leaf pigment levels of two birch species (*Betula pubescens* and *B. pendula*) grown under three different nitrogen levels in outdoor conditions.

## 2. Results

### 2.1. RT-PCR Analysis

**Figure 1:**
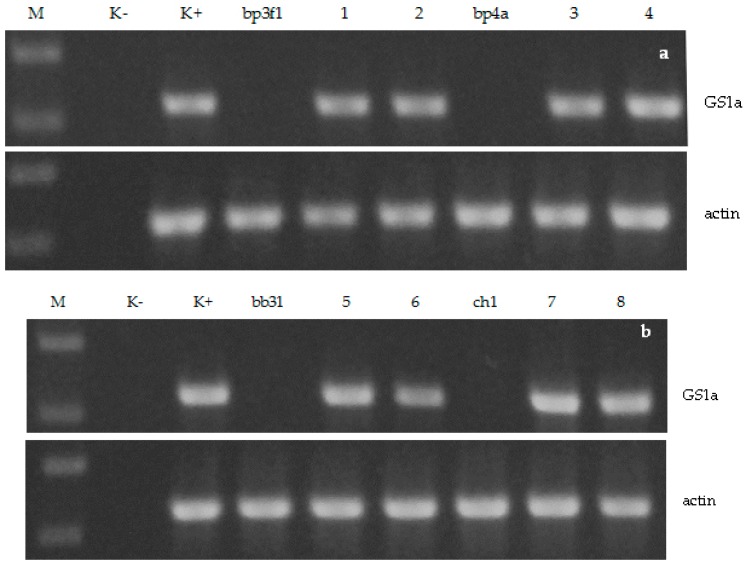
RT-PCR analysis of the GS1a gene expression in *B. pubescens* (**a**) and *B. pendula* (**b**) plants. The actin gene was used as an endogenous control. M—markers (750 and 500 bp); K-—water; K+—pGS (for the GS1a gene) or non-transgenic birch plants (bp3f1 or bb31 for the actin gene); bp3f1, bp4a, bb31, ch1—wild-type plants; 1—F14GS8b; 2—F14GS9b; 3—P9GS18b; 4—P17GS1a, 5—B29GS1; 6—B29GS4; 7—N18GS8a; N18GS8b.

For analysis of the pine GS1a gene expression RT-PCR was performed with total RNA samples extracted from birch leaf tissue. The GS1a transcript was accumulated in all of the analyzed transgenic plants, including nine downy birch-based lines, and five silver birch-based lines. There was no transcription of GS1a gene in the untransformed plants of all genotypes. Results for some of the *B. pubescens* and *B. pendula* lines are shown in Figure 1a,b respectively.

### 2.2. Growth Response of Birch Plants to Nitrogen Availability

Nitrogen supply affected height of birch plants (Table 1). By the end of the vegetation period, in all the control genotypes there were no significant differences when cultivated without nitrogen or in the presence of 1 mM nitrogen, whereas at 10 mM nitrogen plants were 31%–73% higher, depending on genotype. Regardless of the nitrogen availability, *B. pendula* plant heights were approximately 1.5-fold lower as compared to *B. pubescens* plants.

**Figure 2:**
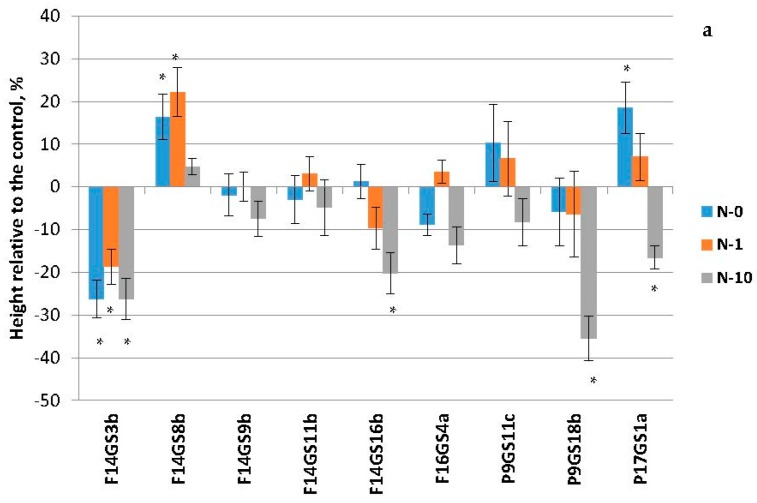

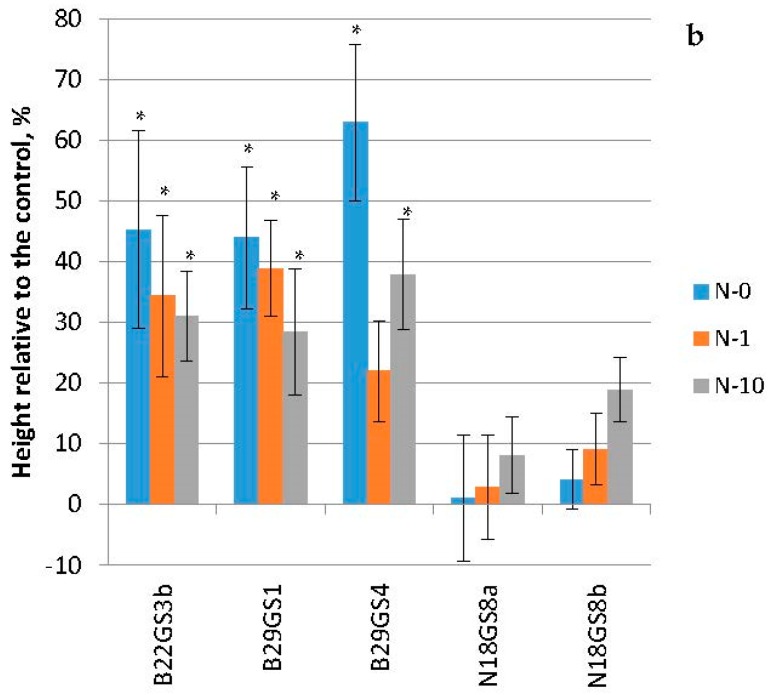
Effect of glutamine synthetase (GS) gene expression and nitrogen fertilization on the growth of transgenic *B. pubescens* (**a**) and *B. pendula* (**b**) plants. Asterisks over single bars indicates that the mean value of transgenic line were significantly higher than that of control plants at 0, 1, or 10 mM nitrogen, respectively, when analyzed by one-way ANOVA (* *p* < 0.05).

In *B. pubescens* species, transformation with the GS1a gene led to a significant increase in heights only in the F14GS8b line (0 and 1 mM nitrogen) and P17GS1a (0 mM nitrogen) (Figure 2a). Plants of other transgenic lines under study, obtained on the basis of both bp3f1 and bp4a genotypes, did not grow as tall as the untransformed plants. Furthermore, the growth difference increased along with the rise of nitrogen availability. On the contrary, all plants of the *B. pendula* transgenic lines were higher than the control ones. However, this effect was significant only for three of five lines, all of which were based on the bb31 genotype (up to 63% in B29GS4 at 0 mM nitrogen) (Figure 2b).

Nitrogen supply had the opposite effect on growth of transgenic plants of *B. pendula* genotypes: the growth difference in bb31-based lines was reduced compared to the control, whereas such difference was increased in ch1-based lines.

### 2.3. Pigment Levels in Leaves of Birch Plants

**Figure 3:**
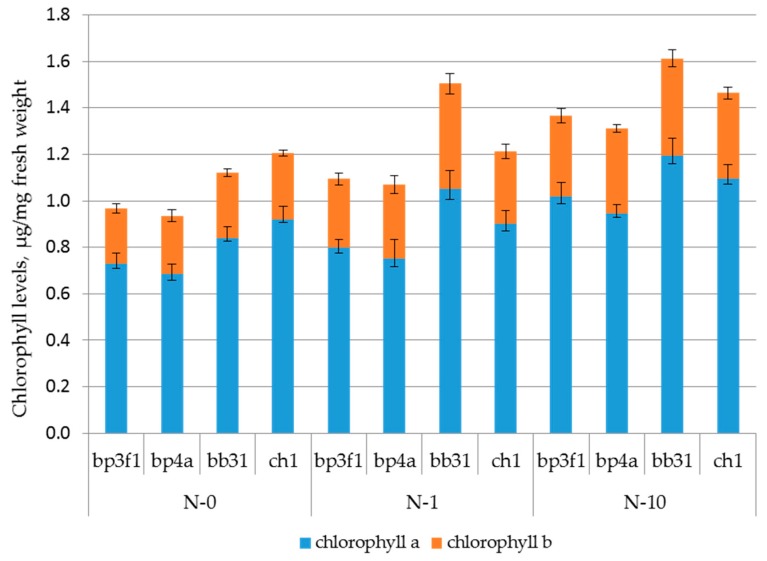
Chlorophyll levels in nontransgenic birch plants. Data bars represent mean ± SE.

Chlorophyll content in nontransgenic plants did not differ among the genotypes of the same species, whereas in *B. pendula* plants there was more chlorophyll as compared to *B. pubescens* (Figure 3). Differences decreased along with an increase in nitrogen availability: *p* = 0.0073 in nitrogen-free variant, *p* = 0.0139 at 1 mM, and *p* = 0.0494 at 10 mM nitrogen. Significant differences in the chlorophyll b content were only observed at 1 mM nitrogen, which was higher in the genotype bb31 as compared to others. Carotenoid content varied from 0.285 to 0.355 μg/mg fresh weight and did not depend on the plant species and nitrogen availability (data not shown). Transfer of the GS gene did not affect the pigment levels in leaves—not a single transgenic line was different from the corresponding control.

Increased nitrogen availability led to an increase in pigment levels in birch leaves. However, this effect was dependent on the plant transgenic status (Table 2). In the response to nitrogen supply, levels of chlorophylls a and b increased in three out of four birch genotypes (except for ch1). However, in most of the transgenic lines of these genotypes nitrogen fertilization did not affect the chlorophyll content. ch1 genotype behaved differently: nitrogen supply almost did not affect the pigment levels in the control, whereas pigment levels were changed in transgenic plants.

## 3. Discussion

Birches are widely present in temperate forests, but they are relatively poorly studied compared to *Populus* species. In Europe, two commercially important birch species occur naturally: silver birch (*Betula pendula* Roth) and downy birch (*Betula pubescens* Ehrh.) [11]. However, to our knowledge, these two species were never compared for their reaction to nitrogen availability. The areas of these species overlap, but *B. pubescens* grows in moist sites and it is more resistant to the cold and occurs further to the north than *B. pendula*, which prefers warmer and drier sites [15].

Our studies have demonstrated that both species responded similarly to nitrogen supply: fertilization with solution containing 1 mM nitrogen was shown to marginally increase plant height, however, 10 mM nitrogen led to more significant increases in growth rates, with −37%–67% and 23%–35% increases for *B. pubescens* and *B. pendula*, respectively. Silver birch genotypes, in addition to displaying a less significant increase in growth rate, also demonstrated lower absolute height in comparison to downy birch (Table 1). Results of known studies of the nitrogen effect on birch growth have not been explicit. Use of fertilizers is not a common practice in the management of birch stands and several attempts made under field conditions in Finland have shown only a weak growth response to fertilization [11]. Studies under greenhouse conditions have shown that nitrogen fertilization had significantly increased the biomass of both *B. pubescens* [16] and *B. pendula* [17], however, a two-year experiment under open-field conditions showed that nitrogen supply significantly increased height of *B. pendula* plants only during the second year, but that the effect during the first year was insignificant [18]. As such, perhaps the effect of fertilization is more prominent under greenhouse conditions.

Significant differences between the plants of the two birch species transformed with a gene encoding the pine cytosolic GS were demonstrated. Whereas all the transgenic silver birch plants demonstrated an increased height in comparison with the nontransgenic control at all nitrogen regimes, only plants of certain downy birch lines were higher than control plants, and only in experiments with low nitrogen availability (0 or 1 mM (Figure 1)). In the work of Gallardo et al. [6], all the 22 lines of hybrid poplar carrying the GS gene were higher than the control following six months of growth in a greenhouse without fertilization. We observed a clear tendency for the reduction of plant heights in comparison with the control along with the increase in the nitrogen availability for all the transgenic lines except for the ones obtained on the basis of the *B. pendula* ch1 genotype. Apparently, additional copies of the GS gene provided enhanced nitrogen recycling in plants during conditions where there was insufficient uptake from the soil. However, under conditions of nitrogen abundance they became an obstacle. Man et al. [19] previously noted that hybrid poplars carrying GS were able to increase their height by 81% at 0.3 mM nitrate, whereas at 10 mM the heights only increased by 35% as compared to the control. In a study by Fuentes et al. [20], tobacco with the GS1 gene grew better than the control only in the absence of nitrogen, and the authors proposed that increased GS activity promoted re-assimilation of photorespiratory ammonium and recycling of other nitrogen-containing compounds. This is an advantage for plantation forestry, as plantations are commonly established on infertile soils. The response of transgenic birch plants to nitrogen availability was genotype-dependent: ch1 genotype based lines differed from other genotypes. It was already shown that genotypes of *Populus tremula* and *P. tremula* × *P. tremuloides* are very different in their reaction to nitrogen fertilization under open-air conditions [21].

Chlorophyll levels in leaves may be used as an indicator of the plant nitrogen status [22]. However, to the best of our knowledge, studies of correlations between chlorophyll levels and nitrogen availability have never been carried out in birch. Overexpression of the GS gene did not change the pigment levels in the leaves of the transgenic plants. Hybrid poplar plants with the GS gene also did not differ in the chlorophyll content from the control at 10 mM nitrogen, whereas at 50 mM levels of chlorophylls a and b significantly increased in the most upper leaves [23]. The chlorophyll content was significantly higher in silver birch plants by approximately 20% (Figure 3) regardless of the nitrogen availability. These data are partially consistent with the results of Li et al. [24], where it was shown that when grown on nutrient-deficient soils *Populus popularis* contained less photosynthetic pigments in its leaves as compared to *P. alba* × *P. glandulosa*, which usually grows on relatively fertile soils. However, these differences were only significant in the treatment without nitrogen, whereas nitrogen fertilization made the difference insignificant.

Our studies have demonstrated that untransformed plants had significantly increased chlorophyll levels (chlorophyll a, b, and their sum) in response to nitrogen supply (only total chlorophyll for *B. pendula* ch1 genotype) (Table 2). In similar studies, nitrogen fertilization increased chlorophyll levels in *Populus balsamifera* ssp. *trichocarpa* × *deltoides* [2], *P. simonii* [25], and *P. deltoides* [26], but did not change pigment levels in *P. alba* × *P. glandulosa* [24]. The differences between bb31 and ch1 genotypes of silver birch that we have shown are rather typical for different species. As in the case of the growth, most of the transgenic lines demonstrated less pronounced responses to nitrogen supply: in contrast to the control plants, differences between chlorophyll levels were insignificant.

Using nitrogen fertilizers to improve productivity is not an optimal solution: first, major parts of them are not assimilated by plants and thus pollute the environment; second, their abundance may disturb normal metabolism, for instance, nitrogen fertilization of *B. pubescens*, in addition to increasing biomass, reduced the concentration of most of the phenolic compounds which may result in reduced resistance against herbivores and pathogens [16]. It was also shown that along with increased inputs of nitrogen, the height and diameter increment of *Betula pendula* decreased [27]. Our data have shown that transgenic birch plants carrying the GS gene have increased nitrogen use efficiency under nitrogen deficient soil conditions. Additionally, we observed significant differences between transgenic downy birch and silver birch in response to nitrogen supply. Such trees with improved efficiency of nitrogen utilization are likely to be especially valuable for the establishment of forest plantations, for which poor soils are usually allocated, in order to avoid competition with agricultural crops.

## 4. Materials and Methods

### 4.1. Plant Material and Growth Conditions

The following four genotypes of two birch species were used in the study: *Betula pubescens* Ehrh. (bp3f1, bp4a), *Betula pendula* Roth (bb31, ch1), and transgenic lines obtained on their basis that contain the pine glutamine synthetase gene. Transgenic plants of *B. pubescens* were obtained via *Agrobacterium*-mediated transformation of birch leaf explants by pGS vector carrying the GS1a gene of *Pinus sylvestris* under the control of CaMV 35S promoter, and their status was confirmed by PCR [12]. Gene transfer and molecular analysis of *B. pendula* plants were carried out following the same procedure. Plants were micropropagated, transferred in a greenhouse at the beginning of April, planted into 1 liter pots with peat:perlite (3:1) at the end of May, and transferred outdoors. In total, 4 control genotypes and 14 transgenic lines (20–30 plants in each group) were planted. During nine weeks (from mid-June to mid-August) the plants were fertilized daily with solutions of macro- and micronutrients containing 0, 1, or 10 mM nitrogen in a calcium nitrate form (100 mL per plant). At the end of July, expression via RT-PCR and pigment levels were analyzed in the leaves from the middle part of the plants. Plant heights were measured once every two weeks during vegetation period.

### 4.2. RT-PCR Analysis

Total RNA was extracted from leaves of birch plants as described by Chang et al. [28]. RT-PCR reaction was carried out as previously described [14]. Information about (i) cDNA nucleotide sequences, (ii) location of primers, and (iii) length of amplicons are presented in Figure S1 (for the GS1a gene from *P.sylvestris*) and Figure S2 (for the Actin gene from *Populus tomentosa*).

### 4.3. Leaf Pigment Analyses

Chlorophyll and carotenoid contents were analyzed using the methods of Wellburn [29]: after pigment extraction with 80% acetone, optical density was measured at 663, 646, and 470 nm wavelengths (Shimadzu UV-1800, Kyoto, Japan).

### 4.4. Statistical Analysis

Statistical analysis was carried out using Statistica 10 software (StatSoft, Tulsa, OK, USA).

## Figures and Tables

**Table 1:** Effect of nitrogen availability on growth (cm) of nontransgenic birch plants.

Species	Genotype	Nitrogen, mM		
		0	1	10
^1^ *B. pubescens*	bp3f1	67.1 ± 2.3 b ^2^	64.0 ± 1.7 b	87.8 ± 3.2 a
	bp4a	52.3 ± 2.8 b	54.0 ± 3.9 b	90.3 ± 2.6 a
*B. pendula*	bb31	40.9 ± 3.8 b	44.2 ± 2.2 b	59.5 ± 6.1 a
	ch1	39.4 ± 2.3 b	41.7 ± 2.5 b	51.4 ± 2.2 a

^1^ Data indicate mean ± SE; ^2^ Different letters in a line indicate significance of differences according to the Duncan test at *p* < 0.05.

**Table 2:** Statistical relevance of the effect of nitrogen availability on pigment levels in birch leaves.

Species	Genotype	Chlorophyll а	Chlorophyll b	Carotenoids	Chlorophylls a + b
*B. pubescens*	bp3f1 (control)	*** ^1^	*	ns ^2^	**
	F14GS3b	ns	ns	ns	ns
	F14GS8b	ns	ns	ns	ns
	F14GS9b	*	ns	ns	ns
	F14GS11b	ns	*	ns	ns
	F14GS16b	*	ns	ns	ns
	F16GS4a	**	**	ns	**
	bp4a (control)	**	*	ns	**
	P9GS11c	*	*	ns	*
	P9GS18b	ns	ns	ns	ns
	P17GS1a	ns	ns	ns	ns
*B. pendula*	bb31 (control)	**	**	*	**
	B22GS3b	ns	ns	ns	ns
	B29GS1	ns	ns	ns	ns
	B29GS4	*	*	*	*
	ch1 (control)	ns	ns	ns	*
	N18GS8a	*	ns	ns	*
	N18GS8b	**	ns	**	**

^1^ Asterisks indicate significant differences between 0 and 10 mM nitrogen, when analyzed by one-way ANOVA (* *p* < 0.05; ** *p* < 0.01; *** *p* < 0.001); ^2^ ns = not significant.

## Supplementary Materials

The following are available online at www.mdpi.com/2223-7747/6/1/4/s1, Figure S1: Nucleotide sequence of cDNA encoding the GS1a gene from *Pinus sylvestris* (X69822), Figure S2: Nucleotide sequence of cDNA encoding the actin gene from *Populus tomentosa* (GQ988367.1).
