# Supplementary material for: Influence of Nitrogen Availability on Growth of Two Transgenic Birch Species Carrying the Pine GS1a Gene
Source: Plants (Basel). 2017 Jan 6;6(1):4. doi: 10.3390/plants6010004 (PMC5371763; doi:10.3390/plants6010004)
Supplement: Supplementary file 1 [file plants-06-00004-s001.pdf]

Plants **2017**, *6*, 4; doi:10.3390/plants6010004

Supplementary Materials: Influence of Nitrogen Availability on Growth of two Transgenic Birch Species Carrying the Pine GS1 Gene

Vadim G. Lebedev, Nina P. Kovalenko and Konstantin A. Shestibratov

1 TTCCCTTCCTCTGGTTTGTTTTGGAGAGTGGCCATGTCGAGCGTATTAACAGACCTTCTC

61 AACCTTGACCTGAGCGACGTGACAGAGAAGGTCATTGCAGAGTATATATGGATTGGAGGA

121 TCAGGAATGGATATGCGCAGTAAAGCCAGATCTCTGTCAGGACCTGTGAGTAGCGTTAAA

181 GAGCTTCCCAAATGGAACTATGACGGCTCCAGCACTGGACAGGCTCAAGGACATGACAGC

241 GAAGTCATTCTATATCCACAAGCTATCTTCCGTGATCCATTTCGCAGAGGAAAGCACATT

301 TTGGTAATCTGTGATGCCTACTCTCCTAATGGGACTGCTATTCCTTCCAACAAGAGGGCT

361 GCAGCAGCGAAAATTTTTAACGAAAAGGCGGTTAGTGATGAAGAGACATGGTACGGGCTT

421 GAACAAGAATATACACTGTTGCAAAAGGACGTCAAATGGCCTCTTGGCTGGCCAATTGGT

481 GGCTACCCCGGTCCTCAGGGCCCATATTACTGTGGAGTTGGAGCTGACAAAGCCTGGGGA

541 CGAGACATTGTTGATGCCCATTATAAGGCTTGTCTCTATTCAGGAATCAATATCAGTGGC

601 ATCAATGGAGAAGTCATGCCAGGGCAGTGGGAATTTCAAGTAGGTCCGTCAGTGGGTATC

661 TCAGCAGCAGATGAGCTGTGGTGTGCTCGTTTTATTATGGAGAGGATTACAGAAAAGGCG

721 GGTGTCGTTCTGTCCTTTGATCCCAAGCCAATTGAGGGGGACTGGAATGGTGCTGGATGC

781 CACACAAATTACAGCACCAAGTCCATGCGCAAGGAGGGAGGCTTCGAAGTAATTAAGAAA

841 GCAATAGAAAAACTGAAGTTGAGGCATAAGGAGCATATTTCTGCCTATGGGGAGGGAAAT

901 GAGAGACGCCTCACTGGTCGGCACGAGACAGCAGACATGAATACCTTTTCCTGGGGTGTT

961 GCAAATCGAGGAGCTTCAGTTAGAGTGGGCCGGGACACAGAAAAAGAAGGAAAAGGTTAT

1021 TTTGAGGACCGTCGACCTGCTTCAAACATGGATCCATACATAGTGACTTCTATGATTGCT

1081 GAGACGACCATTCTATGGAAACCTTAAATTACAAAGTGGAGGCCAGTTACACGCGTGGTC

1141 GTGGTGCTTTGCTTTGGAGGCCAGCGTCACTGATAAGCTAATATGTATGTAAATGTGATG

1201 CCAATGTTTAAGTAGGTTGGTAACTTTGCTTTGGTTGTGGGTAGACCTGAACTTTGGTCA

1261 AACAATTTCCTCTTGCTATATGGATATATATATATTTTTGTATTTGTTCTACTTGTAATA

1321 TGGCGAGGGCTTTAAAAGACTCTCTTTTACCTTTATTTATTCCGTTGTGGAAGATGTATT

1381 CGACAAATTGTTTAGAATGTTTGAATATGATATATTCTTTGTG

Figure S1. Nucleotide sequence of cDNA encoding the GS1a gene from *Pinus sylvestris* (X69822). The location of primers within the sequence is indicated by a straight line for the GS up1 primer and by dotted line for the GS low1 primer. Size of amplicon is 553 bp.

1 CAATGAGCGTGACGCGTCATGGGGTCCGTAGATCAGATACTGAGAACCAACGGCCAAGAT
61 CATTTCAAAGAAGATTTGAGGTTCATTTAAGCTGAATTTCATTTGAGGCTGAAATTTCAT
121 TTCACATCTCCCCCTTTTTTAAAACTAGAGGGCGGGTACCTTGAACAGACGCCCACACAG
181 AGATCGAATCCAGATTTCTAAACGGTATTTCATTTGAGTTTCATTATTTTCTTTGTTAAT
241 CTGGGGTTAGCTTAGAATCCAGCATTATCGATTCAAACAATTAAAAGAATTTATCTTTAA
301 ATGTGGGCCAATTCAGTTTTGTAATTCAGCATTGCTAAGGTCTTGATCTTTGATGTGATT
361 TTATCAAAATGAATATTCAGATTTTGATTGTTACTTGTAAAAAGGAAATGGGATATTTTT
421 ATTTTGCTTTCTTCTTGGATGTTTTTTTGTCAAAATAGTACATTAGATTGTTGTGATTCC
481 CTATTTTTACAGCATTTTAATGTAAAGTTGGCATTTTTAATGTGGCAGGTGAGAGGAAAT
541 GGCAGAAAGTGAAGATATTCAGCCTCTTGTCTGCGACAATGGTACCGGAATGGTCAAGGT
601 ATTAAGCTAGATTCATTTGTGATCTATTTTGGAGTTTAAAGATTCGATTCTTAAAGCGAT
661 TTTGGTAGTGATTGCTGATTTGGGATGGAAATTTTGTAGGCTGGGTTTGCCGGAGATGAT
721 GCACCAAGGGCTGTTTTCCCTAGCATTGTGGGTCGCCCACGCCACACCGGTGTGATGGTT
781 GGTATGGGTCAAAAGGATGCGTATGTTGGTGATGAGGCACAATCAAAGAGAGGTATTTTG
841 ACTTTGAAATACCCAATTGAGCATGGTATTGTTAGCAATTGGGATGATATGGAGAAGATT
901 TGGCATCACACCTTCTACAATGAGCTCCGTGTGGCTCCTGAAGAGCATCCGGTTCTCCTT
961 ACGGAAGCTCCTCTTAACCCCAAGGCCAATCGTGAGAAAATGACCCAGATCATGTTTGAG
1021 ACCTTCAACACTCCCGCAATGTACGTTGCTATCCAGGCTGTCCTTTCCCTTTATGCCAGT
1081 GGTCGTACAACTGGTAAGTGATACAATCCATTTAGTATCTGCTTTTTGATCAAATTCATG
1141 CATGTTAAAACGTTGTTATTGGTTTATTGCATTTGCGAGTTTTCAAAATAAAATGGAGTT
1201 GTACTAATTGATTGATAAACAAAATATTTGGGAAAAAAACACATCTAGATCATGAAAAAA
1261 AATGAAATGTGTTACTGGGAACTTGTCAAGCCCTCAATATTTCTAACTTGTAAGTTGGCA
1321 CAACGTATTTCACCCTTTGTCACAACCACAGTTCGTTTTGGAAATTTTATATGATATCTG
1381 CTGGGATCATGATCATGAAAACCAAAGAAGTTCCTTTAACAACGAAAACCAAAGAAGTTT
1441 CTTCAACAAGCATATAGTTTATCCAGCTTGGTGATTTCCATCTGGACTGTTTAATTATAG
1501 AGATAAATGATATGAATTGATACTTTTCACTATCAATGTGTTTTGGCTAGGTATTGTTCT
1561 GGACTCTGGAGATGGTGTGAGTCACACAGTTCCCATCTATGAAGGCTATGCCCTCCCACA
1621 TGCCATTCTGCGTCTTGACCTGGCAGGCCGTGATCTCACTGATGCCCTAATGAAAATCTT
1681 GACTGAACGTGGCTACTCCTTCACGACCACTGCAGAGCGTGAAATTGTAAGGGACATGAA
1741 GGAGAAACTAGCCTACATTGCTCTTGATTATGAGCAAGAGCTGGAGACAGCAAAGACCAG
1801 CTCATCTGTTGAGAAGAGCTATGAGCTTCCAGATGGGCAGGTTATCACCATTGGAGCTGA
1861 AAGATTCCGTTGTCCAGAGGTCCTCTTCCAACCATCCATGATAGGGATGGAAGCTGCTGG
1921 CATACATGAAACAACATACAATTCTATCATGAAGTGTGATGTTGATATTAGGAAGGATCT
1981 CTATGGCAATATTGTTCTCAGTGGTGGCTCTACTATGTTCCCAGGCATTGCTGATAGGAT
2041 GAGCAAGGAAATTACAGCATTGGCTCCCAGTAGCATGAAGATTAAGGTGGTTGCACCACC
2101 AGAAAGGAAGTACAGTGTCTGGATAGGAGGCTCCATTTTGGCATCCCTCAGCACCTTCCA
2161 GCAGGTACTGCGTCTATTTCTTATATATTTGGGCAGTAAACAGAAGCGGACTTTAAACCT
2221 GATTCTGCACATTTTGATGTCTTCATTGTTATGCCTGTGTTGTATTGTTTAGCATCATTA
2281 TGTGTTAAATGCTTAACAATATGAAATTTTAATTGCAATGATTGGAAGGAGTTACTAGAA
2341 TAATAGTGAAGCTTCAGATGGACATTAAGTAGTTAGAAGTTATATCTGAATTCATGATCA
2401 GGGACGACACTTTTGGACTGTGCTTTTCTAATATGGTTGGAGAAACTTGCTTTCTTTTGA
2461 GTGCAGATGTGGATTGCAAAGGCAGAGTATGATGAGTCCGGTCCATCAATTGTCCACAGG
2521 AAGTGCTTCTAATCGTGCAAAGCAAGAGGCTATTGGTCAAAAACATACTTTATACACTTT
2581 GTAGTACAAAAGGCTTCTAATCAAGTGGTTGCAAAACTATCTTCTGCATTTCGTTACCTC
2641 GTTTGCTCTTTCAACTTGTGTTTTGCTCTGTCAAAGTCTGGAC

Figure S2. Nucleotide sequence of cDNA encoding the actin gene from *Populus tomentosa* (GQ988367.1). The location of primers within the sequence is indicated by a straight line for the Actin 1 up primer and by dotted line for the Actin 1 low primer. Size of amplicon is 561 bp.
